# Hospital and Institutional News

**Published:** 1915-10-23

**Authors:** 


### HOSPITAL AND INSTITUTIONAL NEWS.

### THE EXECUTION OF MISS CAVELL.

If any act were capable of increasing the horror and detestation of the world for German barbarity, surely the execution of Miss Cavell would suffice. The allegation against her was that she had harboured and assisted to escape various prisoners of war. We know enough of German methods to doubt whether the unfortunate lady received a fair trial, or anything but a travesty of one: and even if her guilt of the offences charged were undeniable, we hope we are right in believing that no nation but the German would have regarded execution as a fitting punishment. Miss Edith Cavell, who was nearly fifty years of age, was trained at the London Hospital, and was appointed matron of the training school for nurses opened in 1907 in Brussels under the name of L'Ecole Belge d'Infirmières Diplômées. In September of last year Miss Cavell had the option of being repatriated with some seventy other English nurses, but she declined to leave, and remained at her post.

### THE NORWICH VACANCY.

We regret to have to report that the Board of the Norfolk and Norwich Hospital do not consider that the prestige and administrative efficiency of their hospital will suffer by declining to pay an initial secretarial salary of more than £250, with board and lodging. The remuneration, which is that of a clerk and much less than the remuneration often given to a private secretary, must make it impossible for really competent men of from thirty to fifty years of age to accept the appointment. The chief inducement for a younger man to take such a post is that he would have an opportunity under favourable conditions to show the stuff he is made of and to acquire a position for himself in the hospital world. The decision arrived at by the Norwich Board to offer only £250 a year may lead the careful to the conclusion that the Board at present do not seek the services of a really competent and experienced man. If this be the case, and members of the Board are ambitious to act as superintendent and confine their secretary mainly to the office and clerical work, we can understand the present position. To claim that the rate of remuneration for the secretaries of some other institutions is less than that at Norwich is to show that the standard aimed at by the Norwich Hospital Board is not of the highest. If this be so, they cannot realise the probable effect which their present attitude may have upon the future progress and position of the Norfolk and Norwich Hospital. What we fear is a set-back and a policy which does not make for progress. What are the views of the hospital's governors and the people of Norwich?

### THE RECRUIT'S HEART.

A memorandum for the guidance of medical officers performing recruiting duties has been drawn up by Sir James Mackenzie for the War Office. The points touched on are, of course, connected with Sir James Mackenzie's special study, the heart. He points out that the healthy heart in the young may exhibit murmurs: these are not necessarily indicative of valvular disease. Such physiological murmurs are systolic, and may be found at any part of the præcordium: they are negligible if there is no cardiac enlargement, and if the man's history shows that he can undertake without distress any severe muscular work or play. Nor need a candidate be rejected for extra systoles, if in all other respects the heart appears healthy. It is pointed out that the mere excitement of being examined is enough to set some men's hearts palpitating violently: rest, lying down for a few minutes, will eliminate this excited action. All this is true enough; and it is quite right that medical officers should be warned against the possibility of rejecting a man through ignorance of the facts, for the Army thus loses a recruit, and the man may become hypochondriacal through a baseless anxiety about himself. But in practice, we fancy, the tendency is rather for medical officers to pass men as fit who are not so: and it will be a great pity if this memorandum encourages such laxity by providing a ready-made excuse ("functional murmur") for those who scamp their examination of recruits.

### WHAT IS A WAR HOSPITAL?

The distinction between a war hospital and a military hospital is emphasised in the information published concerning the transformation of St. Luke's Hospital, Horton Green, Bradford, into an institution for the benefit of wounded soldiers to be brought there for treatment from the Dardanelles. With its accommodation for 550 men, and the number of convalescent homes to be attached to it, so that the average stay may be limited to three weeks per patient, a large staff is required. Mr. J. Phillips, who has received the rank of lieut.-colonel, is appointed administrator. In addition to the medical staff under him, the staff comprises a matron, twenty-two sisters, and sixty-eight staff nurses. The new institution is a monument to the patriotism of the Bradford Guardians and other local bodies who have made it possible by taking over the tuberculosis patients now discharged from St. Luke's, many of whom, we understand, have been removed to Barnsley. Mr. R. S. Dawson, chairman of the Bradford Guardians, has lately paid a deserved tribute to the co-operation which local authorities have shown. St. Luke's is a war hospital, not a military hospital, he lately explained, because it is to be administered entirely by the Guardians, whose responsibility therefore is great. The military hospital, of course, is under military control.

### MANCHESTER'S NEW HOME FOR EPILEPTICS.

Considerable discussion has arisen over the projected extension of the Langho Home for Epileptics, which the Manchester Guardians are announced to have decided on. It will be recalled that approximately twenty years ago the Manchester and Chorlton Boards of Guardians established the Langho Home for the benefit of patients within the area controlled by these authorities. Prestwich Guardians did not enter into the scheme, but as time went on the joint committee entrusted with the administration of the institution allowed patients from other Unions, not merely local ones either, to be admitted. Naturally this policy brought forward the question of extension, which was opposed or supported according to the favour or criticism the previous policy had evoked. The amalgamation of the Manchester, South Manchester, and Prestwich areas six months ago has led to a revival of the scheme, which might have gone through had not the action of the Treasury, in refusing permission to local authorities to borrow in the case of all schemes which can be postponed till the end of the war, led to the prospect of a heavy increase in the rates if and when the extension be proceeded with. What, then, is urging the Guardians to proceed with the scheme? and what benefits do they receive in return for extending the right of admission to Unions in remote parts of the country?

### TROUBLE AT THE ENDSLEIGH PALACE HOSPITAL.

Last week it transpired that the authorities of the Endsleigh Palace Hospital for sick and wounded officers were likely to be faced with trouble owing to some discontent among the nursing staff. It is now public property that the majority of the nurses have resigned their appointments as a protest against the dismissal of the matron. Into the merits of the question as to whether this lady's services were better retained or dispensed with, in the interests of the patients, we do not feel called upon to enter. But there are certain considerations which seem not to have been kept in mind, and are worth briefly stating. The managers of any institution have an absolute right to dismiss their matron, whom they pay, and whom they appoint; they have also the right to ask, though they are not bound to follow, the advice of the medical staff as to her capabilities. One of the matron's duties is to supervise discipline among the remainder of the nursing staff: and a wholesome "down tools" of this kind is not *prima-facie* evidence of a discipline that has been effectively supervised. Lastly, besides their loyalty to their head, the nurses of any institution owe a duty to their patients, and in a war hospital in war-time this applies much more than in any other circumstances. It is said that the staff has been replaced by a new one: to the outside observer it might seem that for one hundred patients a smaller staff than the eighty odd previously engaged would suffice.

### WAR HOSPITAL EXPANSION AT MANCHESTER.

Manchester is justifiably proud of the way in which it is responding to the calls for more beds for the sick and wounded from our Armies. The Manchester Base Hospital was originally designed to accommodate 520 beds. To-day there are twenty-two hospitals in being as part of this hospital base, with approximately 3,780 beds, as well as 154 auxiliary hospitals which can take over 8,000. The 2nd Western (Manchester) Military Hospital in Whitworth Street has recently been inspected by Surgeon-General Sir A. Keogh and General Sir W. H. Mackinnon; Sir Alfred expressed the greatest satisfaction with the institution, and was eulogistic on the services which it has already rendered in receiving nearly 30,000 wounded soldiers. It is claimed by the *Manchester Guardian* that the Manchester Hospital is the largest of its kind in the country, and has received more patients than any other. We do not know whether exact figures of the accommodation in the London area have been published, but we should fancy that the accommodation largely exceeds that attributed to Manchester. However, that may be, we heartily congratulate Manchester on the splendid result of its hospital organisation in responding to the country's call.

### FREE CHOICE OF DOCTOR.

In The Hospital of October 2 (page 7) we expressed the opinion that, owing to the depletion of medical panels in all parts of the country, the principle of "free choice of doctor" by insured persons would have to be modified. This modification has been made sooner than we anticipated, for a new provisional regulation under Section 15 of the Insurance Act has been issued which provides that an insured person whose name is included in the list of a practitioner on the panel who is on November 30, 1915, absent on military service shall not be entitled to select another practitioner at the end of the present year unless, in addition to giving notice to the Insurance Committee in the usual way, he satisfies the Medical Service Sub-Committee that he has "reasonable grounds of desiring to be removed from the list of the absent practitioner." It is probable that even such a slight modification as this would in normal times be resented by insured persons, but we trust that under existing extraordinary circumstances they will accept without complaint an arrangement that is made in the best interests of all concerned.

### THE MENTAL HOSPITALS' ROLL OF HONOUR.

Many more names, unfortunately, have now to be added to the list of members of the staffs of the London County Council's mental hospitals who have laid down their lives in the present campaign. Among them we notice Lieutenant Edgar Faulks, of the R.A.M.C., who was a first assistant medical officer in the mental hospital service, and Lance-Corporal Albert Jackson, of the Yorkshire Light Infantry; Private H. C. Rice, of the West Surrey Regiment; Gunner H. W. Turner, of the No. 1 Depôt 22 Co. Royal Garrison Artillery; and Lance-Corporal William West, of the 10th Batt. Royal Marine Brigade, all of whom were attendants at the mental hospitals. We also notice in the County Council's Roll of Honour Lieutenant Stephen Barry Walsh, of the R.A.M.C., an assistant medical officer in the Public Health Department, who has died of wounds.

### WORKING-CLASS SUBSCRIPTIONS IN WAR-TIME.

The difficult problems arising from the financing of hospitals by working-class subscriptions have often been discussed in our columns. The large committees which are popular among these subscribers, the jealous control they exert over their delegates, the elaborate discussions of technical points on which, perhaps, speakers are not fully informed, these difficulties are well known to secretaries of hospitals in industrial centres. There are times when these difficulties press with peculiar weight, as when, for instance, death or resignation removes a man whose judgment and prestige with the working men have been relied on to smooth away the periodic difficulties which arise. It is inevitable that financial questions under the voluntary system should have an air of uncertainty; but how far is it true to say that a large list of working-men's subscriptions is in the long run the most precarious source of income? At Birmingham the Hospital Saturday Fund has had a good year; but the problems of this class of finance are none the less seen in the fact that some 70,000 men have been lost as subscribers by joining the Colours. This is a good instance of the dislocation to which working-class subscriptions are subject; and hospital finance in war-time is especially worthy of anxious study in the strongholds of working-class support.

### THE COOK'S OPPORTUNITY.

With the general rise in prices from which every institutional committee is suffering, it is interesting periodically to place on record what rate is being paid in different districts for the cost of maintenance. Thus the finance committee of a Surrey Board of Guardians has recently confessed that the cost per head per week has risen from 4s. 3d. to 6s. The instances which were given of increases under various heads included the following: Bread £322, milk £200, and tea £85—a trinity which may well cause anxiety. It is interesting to add that attempts have been made to effect economies by a change of diet, so that the above remarkable figures, which do not represent the only articles affected, show how pressing has been the need for economy. As we have often remarked before, economy should be sought, and can certainly be found, rather in the preparation and serving of food than in a radical change of diet. Now is the chance for the really good cooks to show their value to the institutions.

### DRUG ECONOMY IN POOR-LAW INFIRMARIES.

A statement prepared by the clerk to the Southwark Board of Guardians shows what little effect the war has had upon the supply of drugs to Poor-Law infirmaries. The return covers the last four years, the expenditure on drugs in 1911-12 being £1,748; in 1912-13, £1,673; in 1913-14, £1,707; and in 1914-15, £1,716. The high expenditure in 1912 was owing to the fact that in that year there was a great amount of sickness amongst the poor of the borough, the cost of drugs, etc., in the infirmary in 1912 being £1,302, as against £1,237 in 1915. But while there has been no additional expenditure on drugs there have been increases in other departments, the salaries to the staff having increased by nearly £800, and the sum expended on their rations is responsible for an extra £620. Much of this is due to the war and the additional salaries which have had to be paid to the medical and nursing staffs. The cost per inmate per week has increased from 17s. 2¼d. in 1911-12 to 19s. 7½d. in 1914-15, this being exclusive of the repayment of loans and interest thereon. The drug contracts have been amicably arranged by most of the Metropolitan Boards of Guardians. In the great majority of cases the old contracts have been allowed to remain in force, the contractors being permitted to charge any justifiable advance that may have taken place in any article. These have been considered by the Guardians at the close of the quarter, and have been invariably paid. By this means much inconvenience has been avoided, and the drug contractors have been able to work smoothly with the Boards of Guardians. We can quite understand that by the exercise of economy the Southwark Board has been able to prevent any great advance in its drug bill, but when we consider that many of the most commonly prescribed drugs have increased in price enormously we cannot avoid observing that there may have been some extravagance in the past.

### THE CLOSING OF POOR-LAW INSTITUTIONS.

There are many efforts being made by Metropolitan Boards of Guardians to close some of their establishments with a view to reducing expenditure and overcoming the very difficult problem of getting a sufficient staff. There has been a general diminution in the number of the indoor poor, many of the aged men, who long ago were too old for the labour market, having left when they were called upon to fill the places of younger men who had joined the Colours. But the reductions in numbers have not been sufficient in the great majority of cases to justify the closing of an entire institution, though, when the military authorities took over the whole of the buildings belonging to the Lewisham Board of Guardians, places were found in the neighbouring institutions more than sufficient for the number of persons requiring accommodation. Efforts have been made in Southwark to reduce the wards kept open for inmates in normal times, and at the Newington institution two dormitories have been closed, and efforts are to be made shortly to close another. Bermondsey has not been inconvenienced by handing over the institution in Tanner Street to the military authorities, and it is to be hoped it will never again be opened for the accommodation of the poor.

### HOSPITALS AND THE CHOICE OF DISINFECTANTS.

In view of the vast quantities of disinfectants used in hospitals and the public health service generally, it is important that the authorities concerned should carefully note the results of researches directed to improve these prophylactic agents. In this connection it is interesting to observe that the United States Public Health Department has just announced the results of a study of the subject with a view to recommending an effective disinfectant. A number of substances have been investigated with the object of providing a preparation that should be efficient, cheap, and readily compounded of materials available on the market. The substance which is recommended is made from "pine" oil and emulsified with saponified resin according to a definite procedure, the method being simple. At present the full official report of the experiments which justify the conclusion that this disinfectant is as efficient as the preliminary report suggests has not been published, but it will be interesting to see what is the scientific foundation of the recommendation. Of the various disinfecting substances on the market, emulsified coal-tar products are the most commonly used. Some of these are sold as proprietary articles, with trade names that give but little clue to their nature and efficiency. The better ones, however, have a guaranteed "phenol coefficient," thus placing a definite legal responsibility upon the manufacturer. On the other hand, there are manufacturers who avoid this liability, and the public is often unable, therefore, to select a disinfectant which will be at the same time efficient and economical.

### MEDICAL EDUCATION IN WALES.

Some disappointment is being manifested in Wales over the letter of the Treasury on the subject of grants to the Welsh National Medical School. It appears that the Treasury at present contribute £31,000 a year, a fairly substantial sum; but the authorities of the Welsh University have been petitioning for more. The Treasury answer is that a not very large increase will be granted, but only on condition that the University and Colleges ask for a Royal Commission on reorganisation and management, and agree to accept such action as the Government may take as a result of the subsequent report. The *Western Mail* sees in this conditional support, first, testimony to the value of the educational work performed in Wales; and, secondly, dissatisfaction with the reform scheme which the University authorities themselves propounded some months ago. This scheme is dubbed a "poor thing, a medley of petty adjustments . . . a makeshift," and no surprise is expressed that it failed to impress the Board of Education. Meanwhile Cardiff is faced with a prospect of considerable delay in getting the Medical School into full working order: and at the same time Sir W. J. Thomas is asked to "go slow" with his building schemes on account of the war.

### A MATRIMONIAL SCHEME.

It is announced by the Rev. Ernest Houghton, rector of St. Stephen's, Bristol, that in deference to his Bishop he has resigned any official position in connection with the proposed "League for the Marrying of Wounded Heroes"; and that his curate and his honorary consulting physician have also abandoned the enterprise. While sympathising with Mr. Houghton's motives, we feel that the Bishop of Bristol is right in objecting to the participation of his clergy in a scheme fraught with such chances of ill-assorted union as that in question. That patriotic Englishwomen, acutely anxious to do something for England, should contemplate devoting their lives to alleviating the lot of crippled heroes is both natural and creditable; but we can scarcely believe that the indiscriminate allotment of partners on what is practically the lines of a matrimonial agency is the right way for them to put their wishes into practice. Some time ago a lady advertised in the agony column of the *Times* her willingness to marry an incapacitated officer, her own *fiancé* having lately fallen in the field. Here, too, while sympathising with her intentions, and even reverencing her self-abnegation, we yet doubt whether she was setting about her project in a suitable way. We make these remarks not in any carping spirit, for the spirit of personal service disclosed is wholly admirable, but simply because we are convinced of the drawbacks as well as of the advantages of matrimonial alliances based on the casual introduction of agencies or newspaper advertisements.

### THE POSITION OF R.N.V.R. SURGEONS.

A medical correspondent of the *Morning Post* makes out a very strong case for better treatment of the medical members of the R.N.V.R. He points out that these surgeons devoted time and trouble during peace to making themselves efficient, as well as undertaking certain liabilities in the event of war. Some of them are men holding important hospital appointments: are, in fact, surgical consultants. Yet this deserving class, called out compulsorily at the outbreak of war, is actually junior in rank and standing to temporary surgeons hastily enrolled during the war, some of whom are only just qualified. He vouches for one astonishing case in which a medical student was accepted for service with the Navy as surgeon-probationer. After some time he was allowed special leave to go ashore and sit for his medical qualifications; these achieved, he found himself taking precedence as senior officer to his old teacher! This is an extreme case, of course, but it is said that it is typical of similar injustices.

### THE GRIEVANCE ADMITTED.

The author of the communication states that these gross and flagrant grievances, though admitted by the Admiralty, are so contemptuously ignored that the only resource is to bring public opinion to bear upon the official mind. One way of remedying, in part, the anomaly was suggested to the authorities—namely, that they should promote to the rank of (acting) staff surgeon some of the more experienced and eminent among the surgeons of the R.N.V.R. The Director-General, however, declared that in no case could this be done short of eight years' service. Within a month of this declaration, one of the most junior of the

R.N.V.R. surgeons was given the rank of acting staff surgeon for no better reason than influential connections; naturally, this did not do much to remove the sense of grievance latent among the more senior Volunteer surgeons. If the facts are as stated, the Director-General has been made to look ridiculous by some one in the Admiralty, and he should have protested with sufficient energy to get things remedied. His responsibility, at least, would seem to be clear, though doubtless he was not primarily responsible for this piece of bungling injustice.

### THE ECONOMICS OF UNPAID EMPLOYMENT.

This week two communications have reached us which hospital managers actively interested in the many subsidiary organisations which the war has caused to spring up would do well to ponder. The first emanated from the Chester and District Hospital and Ambulance Requirements Association. It stated: "We are already disposing of some 200 articles a week, *for which we charge only the bare cost of material* [italics ours], and we are willing to supply, on the same terms, any organisation or society which is dealing with our Allies' wounded." The inevitable concomitant of such an appeal was contained in a letter written by Mrs. Barlow, Hon. Secretary of the Home Workers' War Aid Association, of 14 Great Smith Street, S.W. She points out that "many thousands of hospital garments ordered by the hospital authorities and relief committees have been made by women to whom the work means home and food. How this has changed. Our lady workers, in elegant caps and aprons, pay to sit in fine airy rooms and feel they are doing great good by "giving their time to stitching. . . . Unpaid workers have taken the work from those they themselves used to employ." This can hardly be gainsaid. It is not a question of supporting one association against another, but of pointing out to each that the sympathy represented by the one, and the hard facts known only too well to the other, must be brought together if voluntary effort of this sort is not to create more evils than it tries to remedy.

### BELLAHOUSTON HOSPITAL OPENED.

The National Hospital erected in Bellahouston Park, Glasgow, by the Scottish Branch of the Red Cross Society was formally opened on October 14 by the Duchess of Montrose. The need for it was abundantly shown by the fact that eighty wounded arrived before the opening ceremony had begun. The hospital contains 700 beds, and is additional to the 500 already set going at Springburn. In accepting the hospital on behalf of the War Office, General Bourke said there were now 10,000 "first line" beds available in Scotland. The Corporation provided a site of fifteen acres. The buildings have cost about £39,000. Some of the blocks and wards have been provided by different counties: thus Ayrshire and Renfrewshire have each given a block; Dumbartonshire and Fifeshire a ward each; while Stirlingshire and Peeblesshire have contributed beds. In all £51,000 has been subscribed for this hospital, so that the whole expense of building and equipping it has been met without encroaching on the general funds of the Scottish Red Cross. The Chairman of the Construction Committee is Mr. Hugh Reid, who has not only devoted much time and business knowledge, but has also lavished personal attention and private generosity on the scheme.

### CARDIAC DISEASE IN SOLDIERS.

The strain and hardship of active service often lead to the development of cardiac disease, or to the aggravation of disease already present. There is reason to believe that in some cases the pathological changes produced are the result of chronic bacillary infection. An investigation into these cardiac conditions is now being organised by Sir James Mackenzie. A certain number of beds at University College Hospital have been allotted for the reception of such cases, where they can be studied by the aid of the most recent and refined methods. Men trained in these methods, acting under Sir James Mackenzie's directions, are now working in several of the new military hospitals, and the result of their collective labours is likely to throw much light upon the problems of cardiac disease and its etiology.

### THE SOUTHWARK MILITARY HOSPITAL.

Arrangements have been completed for the War Council to take over the infirmary of the Southwark Board of Guardians, which will then be known as the Southwark Military Hospital, for sick and wounded soldiers. After the last battle the Local Government Board was approached by the War Council to see whether the Poor-Law authorities could provide further accommodation. A consultation took place between the officials of the Board and the Chairmen and Clerks of the Southwark and Lambeth Boards of Guardians, and it was decided to offer the Southwark Infirmary to the War Council. The acute cases from Southwark Infirmary have been removed to the Lambeth Infirmary, where beds have been provided for 250 patients, whilst the chronic cases will find accommodation in other institutions of the Southwark Board of Guardians. The staff at Southwark Infirmary will, as has been the case when other similar institutions have been taken over, remain at the infirmary, Dr. H. W. Bruce, the medical superintendent, having the temporary rank of major conferred upon him. The infirmary, at the request of the Guardians, will be known as the "Southwark Military Hospital."

### THE MEDICAL CURRICULUM IN AUSTRALIA.

The latest mails from Australia bring the news that at a special session of the Senate of the University of Melbourne, proposals to shorten the medical course by one year, on account of the war, and to make provision for any necessary rearrangement of the lectures, practical work, and times of holding the examinations, were considered. The proposed temporary regulation, agreed to by the University Council, was submitted by Professor Sir Harry Allen, as follows:

"That the curriculum for the degrees of Bachelor of Medicine and Bachelor of Surgery, during the continuance of the war, be modified, and reduced in length from five calendar years to not less than four calendar years and one term." The motion was unanimously agreed to, as was Professor Masson's motion: "That the Professorial Board should have the power to make any modification of the period of lectures and practical work, or such change in the times of examinations or adjustments of students' fees as may be necessary." Both were ratified by the State Parliament a week later. In Committee an amendment was accepted disallowing non-British graduates from practising on a four years' course qualification. The Victorian Medical Act is to remain in force until a day appointed by the Governor in Council, but not later than six months after the termination of the war.

### EXPLOITING THE MEMORY OF THE DEAD.

A correspondent of influence and standing draws our attention to what he describes as the "disgraceful" form of advertising for money which a certain hospital is now employing. Under the heading "In memoriam," which usually succeeds the obituary advertisements in the daily newspapers, there are, as we all know, some notices "in loving memory" of persons who died a year, or perhaps many years ago. Those who wish to use this column as a channel for appeals insert a notice such as the following: "In grateful memory of ———— who died in ———— a generous benefactor by will of the ———— hospital." It is, of course, the addition of the last eight words which seem so outrageous to our correspondent. The ground of objection, when summarised, is the title of this paragraph. The idea, no doubt, is to induce gifts by holding out to subscribers of a certain sum the prospect of an "In memoriam" notice. Perhaps by a gift of money running into four figures, the hospital would contract annually to publish an "In memoriam" notice of this kind. What suggested this type of advertisement to the secretary we do not know. No one would venture to call it a high-class type of appeal. It has neither principle nor reasoned basis, but is a catchpenny method devised to attract gifts from those whose understandings and sympathies cannot be aroused by facts, needs, or desire for personal service. Catchpenny methods, however, should never be the basis of hospital or any other financial policy. That they are likely to turn away the sympathies of former hospital supporters without attracting other loyal workers in their place is amply proved by the correspondence we have received in condemnation of the criticised advertisement.

### PANEL PRACTITIONERS' ACCOUNTS.

It is common knowledge that, in consequence of the enlistment of large numbers of insured persons from all districts, it is an extremely difficult matter to calculate even approximately the amount of money due to health insurance practitioners, and that therefore they are not being paid the full amount which their lists entitle them to. This matter was the subject of several questions in the House of Commons on October 14. Sir Philip Magnus asked the Parliamentary Representative of the Insurance Commissioners whether he was aware that there was still a large sum of money due to practitioners for their services during the year 1914 and whether he could take steps to accelerate these payments. Mr. Dixon also asked for an explanation of the reasons justifying the deductions insisted upon by the Commissioners of one-third of the quarterly accounts of panel practitioners and institutions after service had been rendered and an agreement entered into with each party; why there had been no advance on account nor adjustment of the deductions of 10 per cent. made in the accounts of doctors and institutions for the year 1914; and if the Government would instruct the Commissioners to deal with the reductions owing to enlistments upon a less arbitrary and more generous basis by a temporary increase of the Treasury grant. The reply of Mr. Charles Roberts to both questions was substantially the same; he referred both Sir Philip Magnus and Mr. Dixon to a reply he had given to the member for Mid-Derbyshire nearly three months ago, which was to the effect that there was nothing arbitrary or ungenerous in the Commissioners' procedure, that the balance due would be ascertained and paid as soon as practicable, and that he could not accept the suggestion that the Exchequer should provide a further grant. While we have full sympathy with health insurance practitioners in this matter, we would point out that the Commissioners are confronted by an extraordinarily difficult statistical problem. It is to be feared that in the case of some practitioners it may be found that, although one-third of the quarterly accounts has been deducted, the final calculations will show that the balance still owing is not substantial.

### THIS WEEK'S DRUG MARKET.

As was inevitable, in view of the developments in the Near East, the price of Turkey opium has again advanced very substantially; the value is now in fact higher than it has ever been at any rate during the last half-century, and there is, of course, little doubt that it will advance still further. Fortunately, a fair supply of Persian opium is available for manufacturers of morphine and codeine, but it would not be surprising if makers of these alkaloids were compelled to raise their quotations. Quinine has continued to increase in price, the value having more than doubled during the past few weeks. Bromides are again dearer, and still higher prices may be asked in the immediate future. Barbitone, phenacetin, phenazone, guaiacol carbonate, iron and quinine citrate are all dearer, and the advancing tendency of salicylic acid and sodium salicylate continues. There is a firmer tone in the quicksilver market, but prices of mercurials are unchanged, although it is still very difficult to obtain deliveries from the makers.

